# Proteomics and Metabolomics Reveal Novel Impacts of Choline Supply on Calf Hepatocytes Experiencing Accumulation During a Fatty Acid Challenge

**DOI:** 10.3390/metabo16070451

**Published:** 2026-06-26

**Authors:** Yaqi Chang, Bin Jia, Yaran Si, Zexin Zhang, Jiachen Liu, Yue Gao, Junhao Wang, Yanhui Wang, Juan J. Loor, Bingbing Zhang, Wei Yang

**Affiliations:** 1 College of Animal Science and Veterinary Medicine, Heilongjiang Bayi Agricultural University, Xinyang Rd. 5, Daqing 163319, China; c15046286438@163.com (Y.C.); jiabin2833@126.com (B.J.); siyaran0903@163.com (Y.S.); zhangzexin1201@126.com (Z.Z.); 13945819054@163.com (J.L.); 15046826828@163.com (Y.G.); wangjunhao05182026@163.com (J.W.); yanhui69lp@163.com (Y.W.); 2 Department of Animal Sciences, Division of Nutritional Sciences, University of Illinois, Urbana, IL 61801, USA; jloor@illinois.edu; 3 College of Life Science and Technology, Heilongjiang Bayi Agricultural University, Xinyang Rd. 5, Daqing 163319, China

**Keywords:** fatty liver, nutrition, transition cow, lipotoxicity

## Abstract

**Background/Objectives:** Exposure to high and sustained levels of non-esterified fatty acids (NEFA) in the peripartal period is the main cause of fatty liver disease in dairy cows. Rumen-protected choline is often fed as part of the nutritional management of peripartal cows, with in vivo and in vitro data indicating positive effects of this nutrient on alleviating liver lipid accumulation. Although hepatic molecular mechanisms associated with choline supply have been studied using a target gene, protein, or metabolite approach, application of high-throughput technologies could vastly enhance fundamental knowledge on the functional role of choline. The main objective was to challenge isolated hepatocytes with a mixture of NEFA and determine proteome- and metabolome-wide effects in response to choline supply. **Methods:** Three healthy female calves (1 d old, 30–45 kg) were sacrificed to harvest hepatocytes. During a 12 h incubation, isolated hepatocytes were challenged without NEFA (control), 1.2 mM NEFA (c9-18:1, 18:2, 16:0, 18:0, and c9-16:1 at 43.5%, 4.9%, 31.9%, 14.4%, and 5.3% of total NEFA, respectively), or NEFA for 6 h followed by 10 μM choline chloride for another 6 h (NEFA + Chol). iTRAQ labeling-based protein profiling and GC/MS-based metabolomics profiling were used to determine changes in proteins and metabolites. Differentially abundant proteins for each group comparison were determined at a threshold of 1.4-fold change. Differences in metabolite profiles were assessed via pairwise comparisons. A subset of differentially abundant proteins was validated via qRT-PCR and Western blotting. **Results:** Compared with the control, there were 90 proteins and 22 metabolites in the NEFA group, and 83 proteins and 29 metabolites in the NEFA + Chol. Compared with NEFA, there were 49 proteins and 17 metabolites in the NEFA + Chol group. Greater abundance of hexokinase-1 (HK1), fructose-bisphosphate aldolase (ALDOA), mitochondrial pyruvate carrier 1 (MPC1), and increased concentrations of lactate with high NEFA treatment alone suggested greater glycolytic and TCA cycle activity. Accumulation of triacylglycerol in the NEFA group was associated with lipotoxicity and markers of inflammation, such as greater abundance of prostaglandin reductase 1 (PTGR1), serious cell autophagy processes, such as greater abundance of cell division cycle 42 (CDC42), and NFκB-related proteins. Choline supplementation reduced TAG partly due to greater VLDL secretion driven by greater abundance of diacylglycerol acyltransferase (DGAT1), perilipin 3 (PLIN3), and apolipoprotein C-III (APOC3). In addition, a greater abundance of carnitine O-palmitoyltransferase 1b (CPT1B) with choline suggested enhanced mitochondrial β-oxidation. Activation of the CDC42/JNK pathway and ROS/NFκB axis-related proteins, along with depressed PI3K/AKT/RAC-related proteins, indicated enhanced mitochondrial autophagy in response to NEFA. **Conclusions:** Overall, data confirmed published effects of choline on TAG accumulation, VLDL secretion, and fatty acid oxidation, while highlighting negative effects of NEFA on the respiratory electron transport chain, autophagy, and inflammatory processes.

## 1. Introduction

Fatty liver disease is one of the most common metabolic disorders in peripartal (transition) dairy cows [1]. Negative energy balance (NEB) due to insufficient dry matter intake to sustain increased energy requirements for maintenance of body functions and milk production leads to mobilization of fat depots, resulting in high and sustained plasma concentrations of non-esterified fatty acids (NEFA) [2]. In turn, flooding of the liver by the ensuing surge of NEFA, along with the inherently low rates of secretion of very low density lipoproteins (VLDL), is the main cause for the development of fatty liver [3].

Choline, a key micronutrient, is essential for phosphatidylcholine synthesis and plays an important role in the packaging and export of triacylglycerol into VLDL [4]. It is well-established in non-ruminants that low choline intake is a cause for the development of fatty liver disease [5]. In the same way, some studies with dairy cows have reported potential benefits of post-ruminal choline supply on liver health and reduction in lipid accumulation [6,7]. However, recent reports with a meta-analysis approach have discussed and highlighted that the direct effects of rumen-protected choline (RPC) in reducing liver TAG are still inconsistent [8,9]. Thus, additional studies at the proteomic and metabolomic level could aid in identifying additional mechanisms involved in the hepatic response to RPC.

Using molecular techniques to study target genes in lipid metabolism-related regulation pathways, previous studies reported positive associations between lower liver TAG in dairy cows and increased abundance of liver carnitine palmitoyltransferase 1 (CPT1), which was associated with stimulation of fatty acid (FA) oxidation, and increased apolipoprotein A5 (ApoA5) and apolipoprotein B (ApoB), which suggested increased VLDL assembly [10,11]. Despite somewhat consistent effects in terms of lipid metabolism, it is unknown what (if any) additional biological effects might be responsive to choline supply, especially when the liver faces an influx of high concentrations of NEFA. Thus, the objective of the present study was to use proteomic and metabolomics profiling to study large-scale changes in profiles in vitro in response to NEFA challenge of isolated bovine hepatocytes alone or in combination with choline.

## 2. Materials and Methods

The Ethics Committee for the Use and Care of Animals, Heilongjiang Bayi Agricultural University (Daqing, China), approved the animal use protocol. The ethical approval number was SMKXJSXY2022003, with the approval date of 10 March 2022.

### 2.1. Hepatocyte Isolation and Culture

Primary hepatocytes were isolated from 3 newborn female Holstein calves (1 d old, 30–45 kg) according to published protocols from our laboratory [12]. Briefly, calves were anesthetized with sodium thiamylal, heparinized intravenously, and euthanized via saturated potassium chloride injection (150 mg/kg body weight; Thermo Fisher Scientific, St. Louis, MO, USA) immediately after caudate lobe removal. The lobe was perfused, cleared of vessels/connective tissue, minced, filtered, and seeded in 6-well plates (1.5 × 10^6^ cells/well) in RPMI-1640 medium supplemented with 10% newborn calf serum, 10^−6^ mol/L insulin, 10^−6^ mol/L dexamethasone, and 10 μg/mL vitamin C at 37 °C with 5% CO_2_. After 5 h, the medium was replaced with growth medium containing 10% newborn calf serum, which was refreshed every 24 h.

### 2.2. Preparation of NEFA Mixture and Treatment

The NEFA (Sigma-Aldrich, St. Louis, MO, USA) used in this study was prepared as described in a manuscript from our laboratory [13], and included oleic acid (10.875 mM), linoleic acid (1.225 mM), palmitic acid (7.975 mM), stearic acid (3.6 mM), and palmitoleic acid (1.325 mM) prepared by diluting individual fatty acids in 0.1 M KOH at 60 °C, and adjusted to pH 7.6 with hydrochloric acid (1 mM).

After 72 h of culture, fresh isolated hepatocytes were cultured for 6 h with RPMI-1640 basic medium without serum, then RPMI-1640 containing 2% bovine serum albumin (BSA) was used. The actual experiment involved a 12 h incubation, during which isolated hepatocytes (6 replicates, 3 biological replicates with 2 technical replications per group) were challenged 4.8 mM KCl (control), 1.2 mM NEFA (c9-18:1, 18:2, 16:0, 18:0, and c9-16:1 at 43.5%, 4.9%, 31.9%, 14.4%, and 5.3% of total NEFA, respectively), or NEFA for 6 h followed by 10 µM ultrapure water dissolved choline chloride (Sigma-Aldrich, St. Louis, MO, USA) for another 6 h (NEFA + Chol).

### 2.3. Cellular TAG Content and Lipid Droplet Fluorescence Assay

Content of TAG in primary hepatocytes was determined using an enzymatic assay kit (Applygen Technologies Inc., Beijing, China) in accordance with the manufacturer’s instructions. Lipid droplets were assessed by fluorescence using cellular staining with BODIPY 493/503 (Invitrogen Corporation, Carlsbad, CA, USA) according to the manufacturer’s instructions. Images were obtained with a confocal laser-scanning microscope (Leica TCS SP8; Leica, Wetzlar, Germany) with 40×/1.3 numerical aperture (NA) differential interference contrast and analyzed with the instrument’s software. Each experimental group had 6 replicates.

### 2.4. Protein Extraction, Digestion, and iTRAQ Labeling

Cells (n = 3 samples pooled per group) were collected and washed with PBS and then scraped in lysis buffer containing 7 M urea, 1.4 M thiourea, and 4% 3-[(3-Cholamidopropyl) dimethylammonio]propanesulfonate (CHAPS). After centrifugation at 14,000× *g* for 20 min, protein concentration in the supernatant was detected with the Bradford assay kit (Solarbio, Beijing, China). One hundred μg total protein from pooled samples in each experimental group was reduced and alkylated by incubating with 5 mM dithiothreitol (DTT) for 60 min, followed by 10 mM iodoacetamide for 40 min. Proteins were washed twice in 200 µL 100 mM Tris-HCl containing 8 M Urea by hyperfiltration at 12,000× *g* for 20 min. The precipitates were collected and washed with 200 mM triethylammonium bicarbonate (TEAB) twice. After centrifugation at 12,000× *g* for 20 min, proteins were digested with trypsin (1:50, *w*/*w*) (Promega, Madison, WI, USA) at 37 °C overnight. The digested peptides were washed twice with double-distilled H_2_O and collected by centrifugation in 40 μL of 0.5 M TEAB.

Twenty μL of protein from pooled samples in each treatment group was used for iTRAQ. iTRAQ labeling was performed according to the manufacturer’s instructions (Applied Biosystems, Foster City, CA, USA). Labeled samples were fractionated using high pH reverse-phase fractionation (RPF) chromatography (Dionex UltiMate 3000 high-performance LC system, Thermo Fisher Scientific, MA, USA). LC-MS/MS analysis was performed on a nano LC-MS/MS system (UltiMate 3000 RSLC nano system, Thermo Fisher Scientific, MA, USA).

### 2.5. qRT-PCR Analysis and Western Blotting

A subset of differentially abundant proteins was evaluated at the mRNA level (n = 6 replicates per group; bold marked in Table 1) via qRT-PCR. In addition, mRNA abundance for selected proteins in enriched pathways related to peroxisome proliferation-activated receptor alpha (PPARα) and phosphatase and tensin homolog (PTEN) was analyzed. Total RNA was extracted using Trizol RNA extraction reagent (Invitrogen Corporation, Carlsbad, CA, USA) from each of 6 replicates per group. The RNA was reverse transcribed into cDNA using Reverse Transcriptase M-MLV (RNase H-) (Takara Bio, Inc., Otsu, Japan). qRT-PCR was performed on an Applied Biosystems 7300 real-time PCR system using SYBR Premix Ex Taq I (F. Hoffmann-La Roche AG, Basel, Switzerland). Gene primers were designed using Primer Premier 5 and are shown in Table A1 of the Appendix A. Gene expression was assessed with the 2^−ΔΔCT^ method.

Western blotting analysis of key proteins associated with insulin and cell growth, or transcriptional control of inflammation, was done using specific commercial antibodies. Protein kinase B (AKT; 1:500, ab18785; abcam, Burlingame, CA, USA) and nuclear factor κB (NFκB) P65 (1:1000; D14E12; Cell Signaling; Danvers, MA, USA) were the target proteins, and their abundance was normalized to β-actin (internal control; 1:1000, sc-47778; Santa Cruz Biotechnology Inc., Santa Cruz, CA, USA). Data (6 replicates per group) were generated with a ProteinSimple imager (ProteinSimple, San Jose, CA, USA). Band intensity was quantified using the ImageJ software (version 1.54, National Institutes of Health, Bethesda, MD, USA).

### 2.6. Metabolomics Analysis

Cells (n = 6 replicates per group) were extracted with 600 µL extraction liquid (methanol:chloroform = 3:1 vol/vol) with 20 μL of L-2- chlorophenylalanine (1 mg/mL stock in dH_2_O) as internal standard. The mixture was homogenized for 4 min at 45 Hz, then ultrasound-treated for 5 min (while on ice water). This process was repeated 5 times, followed by centrifugation at 19,300× *g* for 15 min at 4 °C. Four hundred-fifty μL of the supernatant was transferred to a new GC/MS glass vial, and 12 μL from each sample was pooled as a quality control sample. After the mixture was freeze-dried, 80 μL of methoxyamine HCl in pyridine (20 mg/mL) was added and incubated at 80 °C for 30 min. Then, 40 μL of N,O-bis(trimethylsilyl)trifluoroacetamide (BSTFA) containing 1% trimethylchlorosilane (TCMS, REGIS Technologies Inc., Morton Grove, IL, USA) was added and incubated at 70 °C for 2 h. Samples were run in an Agilent 7890 gas chromatograph system coupled with a Pegasus HT time-of-flight mass spectrometer for GC-TOFMS analysis. The system utilized a DB-5MS capillary column coated with 5% diphenyl cross-linked with 95% dimethylpolysiloxane (30 m × 250 μm inner diameter, 0.25 μm film thickness; J&W Scientific, Folsom, CA, USA). A 1 μL aliquot of the analyte was injected in splitless mode. Helium was used as the carrier gas, the front inlet purge flow was 3 mL/min, and the gas flow rate through the column was 1 mL/min. The initial temperature was kept at 50 °C for 1 min, then raised to 310 °C at a rate of 10 °C/min, then kept for 8 min at 310 °C. The injection, transfer line, and ion source temperatures were 280, 270, and 220 °C, respectively. The energy was −70 eV in electron impact mode. The mass spectrometry data were acquired in full-scan mode with the *m*/*z* range of 50–500 at a rate of 20 spectra per second after a solvent delay of 366 s.

### 2.7. Statistical Analysis

Empirical differences in protein profiles were determined via Proteome Discoverer 1.4 (Thermo Fisher Scientific) using raw files. Mass spectrometry data were analyzed using ProteinPilotTM v4.5 (Applied Biosystems), peptide identification was done using the Paragon algorithm against the UniProt Bos protein database (http://www.uniprot.org/, accessed on 15 October 2022), and the identified protein ID was converted to a UniProt ID and then mapped to Gene Ontology (GO) IDs by protein ID. “Differentially abundant” proteins for each group comparison were determined at an empirical threshold of 1.4-fold change, and were classified according to GO (http://www.geneontology.org, accessed on 15 October 2022) annotation into two categories: molecular function and biological processes. The Kyoto Encyclopedia of Genes and Genomes (KEGG, http://www.genome.jp/kegg/ or http://www.kegg.jp/, accessed on 15 October 2022) database and Reactome Database (https://reactome.org, accessed on 15 October 2022) were used to identify enriched pathways by a hypergeometric distribution test to compare the enrichment of the differentially abundant protein against all identified proteins, and pathways enriched were considered significant at *p* < 0.05.

Data from qRT-PCR and Western blotting were analyzed using SPSS 22.0 (SPSS Inc., Chicago, IL, USA) and GraphPad Prism 7.00 (GraphPadSoftware, San Diego, CA, USA). Statistical significance was determined using one-way ANOVA followed by post hoc Bonferroni correction. Differences were considered significant at *p* < 0.05. Results are reported as means ± standard error of the mean.

Statistical analysis of metabolomics data relied on the Chroma TOF 4.3X software (LECO Corp., St. Joseph, MI, USA, LECO-Fiehn Rtx5 database). The retention time index method was used for peak identification. SIMCA software (V14.1, MKS Data Analytics Solutions, Umea, Sweden) was used for multivariate statistical analyses, including principal component analysis (PCA) and orthogonal partial least-squares discriminant analysis (OPLS-DA). Differences in metabolite concentrations were assessed using multiple testing and corrected by the two-stage-up method of Benjamini, Krieger, and Yekutieli to control the false discovery rate at 0.05, and Variable Importance in the Projection (VIP) of the OPLS-DA model >1. The KEGG (http://www.genome.jp/kegg/, accessed on 15 October 2022) and NIST (https://webbook.nist.gov/chemistry/, accessed on 15 October 2022) were used to search for the pathways of metabolites. MetaboAnalyst (http://www.metaboanalyst.ca, accessed on 1 November 2022), which uses the high-quality KEGG metabolic pathway as the backend knowledge base, is used for pathway enrichment analysis of the significant metabolites.

## 3. Results

### 3.1. Content of TAG and Lipid Droplets

**Figure 1:**
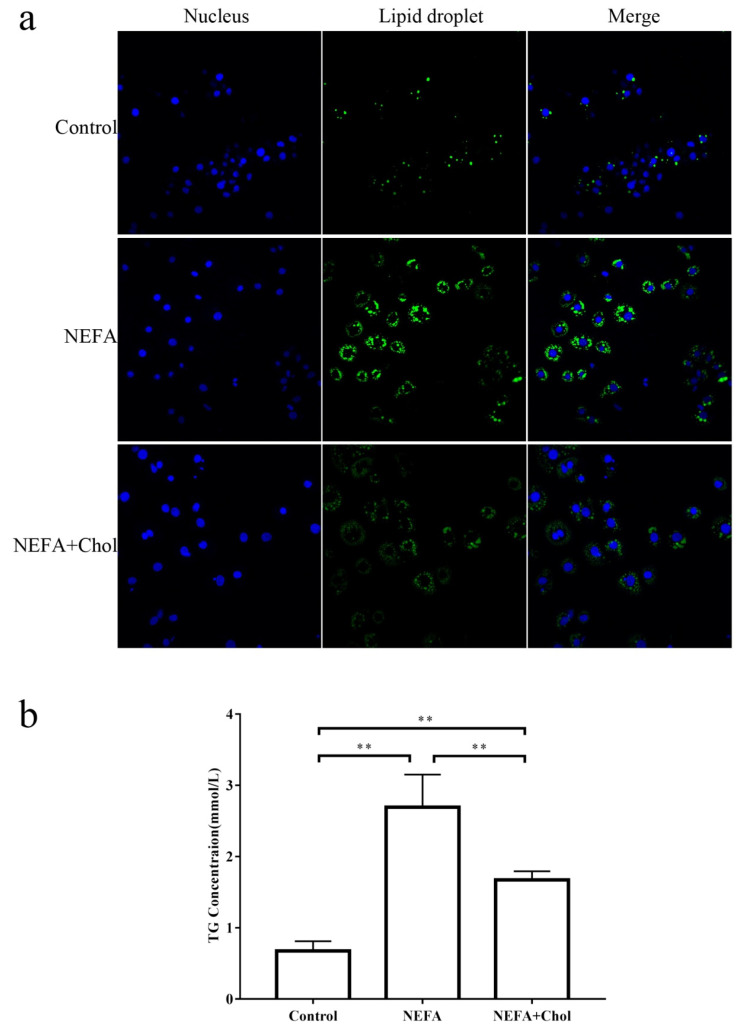
Effects of choline supply on high NEFA-induced lipid accumulation in isolated hepatocytes. During a 12 h incubation, isolated hepatocytes were challenged without NEFA (control), 1.2 mM NEFA (c9-18:1, 18:2, 16:0, 18:0, and c9-16:1 at 43.5%, 4.9%, 31.9%, 14.4%, and 5.3% of total NEFA, respectively), or NEFA for 6 h followed by 10 μM choline chloride for another 6 h (NEFA + Chol). Each assay was performed in 6 replicates. Data are means ± SEM. Lipid droplet fluorescence (Panel (**a**)). Blue fluorescence indicates cell nuclei; green fluorescence indicates lipid droplets. The intensity of green fluorescence increases with the number of lipid droplets in the cell. Cell triglyceride content (Panel (**b**)). ** *p* < 0.01 indicate significant differences.

Compared with the control, hepatocytes in the NEFA and NEFA + Chol group had greater (*p* = 0.0001 and *p* = 0.0028, respectively) TAG concentrations (Figure 1a,b), while the NEFA + Chol group had lower TAG concentrations compared with the NEFA group (*p* = 0.0025).

### 3.2. Differentially Abundant Proteins

**Figure 2:**
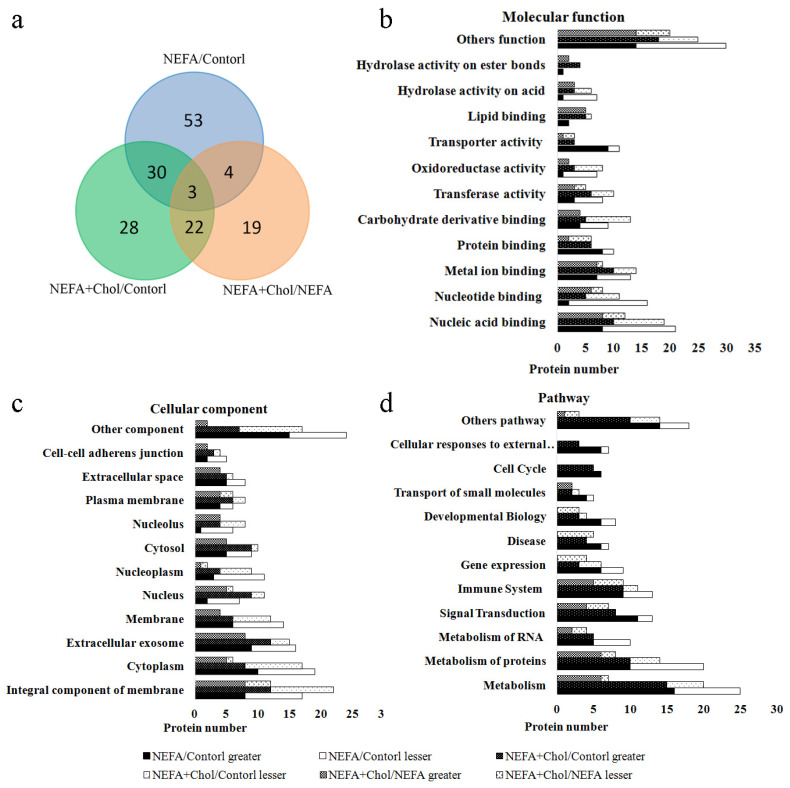
GO term and pathway analysis of greater or lesser abundance proteins. During a 12 h incubation, isolated hepatocytes were challenged without NEFA (control), 1.2 mM NEFA (c9-18:1, 18:2, 16:0, 18:0, and c9-16:1 at 43.5%, 4.9%, 31.9%, 14.4%, and 5.3% of total NEFA, respectively), or NEFA for 6 h followed by 10 μM choline chloride for another 6 h (NEFA + Chol). Correlations of differentially abundant proteins among groups (Panel (**a**)). Molecular functions enriched with differently abundant proteins (Panel (**b**)). Cellular components enriched with differently abundant proteins (Panel (**c**)). Pathways enriched with differently abundant proteins (Panel (**d**)).

There were 2892 proteins identified in each sample in the quantitative proteomic study. Compared with the control, 40 proteins had greater abundance, and 50 had lesser abundance in the NEFA group, and 49 proteins had greater abundance, and 34 had lesser abundance in the NEFA + Chol group. Compared with NEFA, there were 31 proteins with greater abundance and 17 with lesser abundance in the NEFA + Chol group. Among these proteins, as depicted in Figure 2a, a total of 30 proteins were altered in both the NEFA versus control and NEFA + Chol versus control comparisons, 22 in both the NEFA + Chol versus control and NEFA + Chol versus NEFA comparisons, and four in both NEFA versus control and NEFA + Chol versus NEFA. Only four proteins were similarly affected among the three groups.

### 3.3. GO and Pathway Analysis

The GO term and pathway analysis of differently abundant proteins is reported in Figure 2b–d. Molecular functions enriched with differently abundant proteins are involved in nucleic acid binding, metal ion binding, protein binding, oxidoreductase activity, transporter activity, lipid binding, and hydrolase activity. Cellular components enriched with differentially abundant proteins include cytoplasm, extracellular exosome, membrane, nucleus, plasma membrane, and cell–cell adherens junction. Pathways enriched with differentially abundant proteins include metabolism, signal transduction, immune system, disease, developmental biology, transport of small molecules, cell cycle, and cellular responses to external stimuli. Among enriched subpathways (Table 1) were metabolism of lipids, metabolism of carbohydrates, citric acid cycle and respiratory electron transport, metabolism of amino acids and derivatives, signaling by receptor tyrosine kinases, signaling by interleukins, neutrophil degranulation, and RNA polymerase II transcription.

### 3.4. mRNA Abundance for Genes Encoding Target Proteins

**Figure 3:**
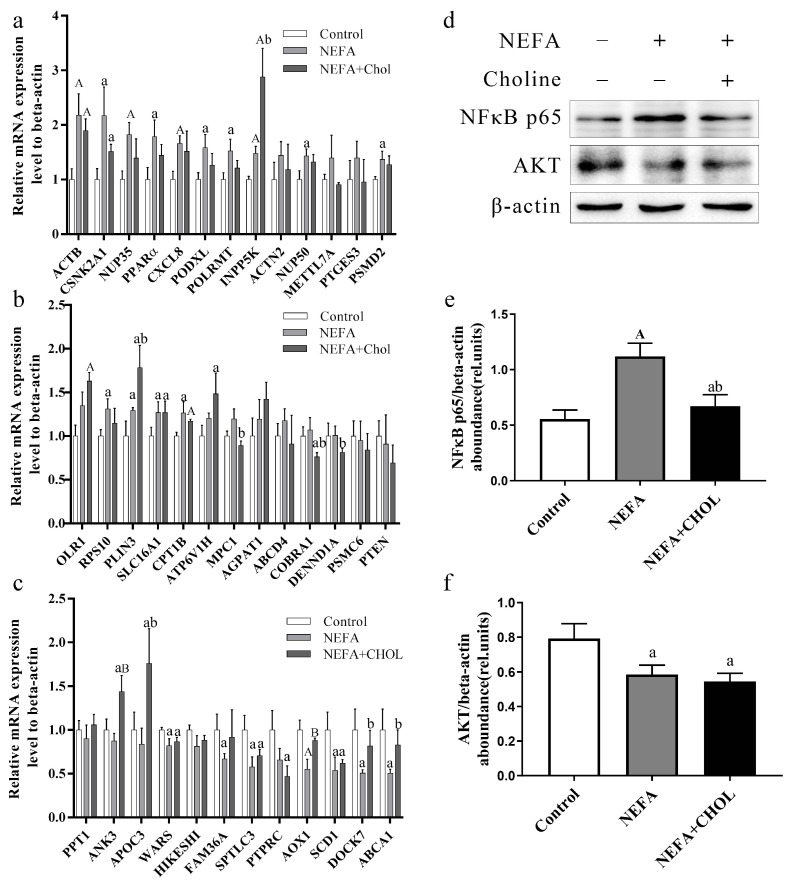
Verification analysis of selected proteins. During a 12 h incubation, isolated hepatocytes were challenged without NEFA (control), 1.2 mM NEFA (c9-18:1, 18:2, 16:0, 18:0, and c9-16:1 at 43.5%, 4.9%, 31.9%, 14.4%, and 5.3% of total NEFA, respectively), or NEFA for 6 h followed by 10 μM choline chloride for another 6 h (NEFA + Chol). Each assay was performed in 6 replicates. Data are means ± SEM. Gene expression analysis of different proteins and PPARα and PTEN (Panel (**a**–**c**)). Protein abundance of NFκB and AKT (Panel (**d**–**f**)). Lowercase letter a vs. control *p* < 0.05, uppercase letter A vs. control *p* ≤ 0.01, lowercase letter b vs. NEFA *p* < 0.05, uppercase letter B vs. NEFA *p* ≤ 0.01.

As shown in Figure 3a–c, the qRT-PCR results indicated that the expression level of most genes was consistent with transcription prediction and protein abundance uncovered by the iTRAQ data. However, 9 genes were inconsistent with transcription prediction according to the iTRAQ data: compared with the control group, neither palmitoyl-protein thioesterase 1 (*PPT1*) in the NEFA + Chol group nor mitochondrial pyruvate carrier 1 (*MPC1*) in the NEFA group had a significant change. In addition, compared with the Control group, 40S ribosomal protein S10 (*RPS10*) and podocalyxin-like (*PODXL*) in the NEFA group were upregulated (*p* < 0.05), and expression of ANK3 protein (*ANK3*) in the NEFA + Chol group was also upregulated compared with the NEFA group (*p* < 0.05). There was no significant difference in *METTL7A* among the three groups. Compared with the control group, DNA-directed RNA polymerase (*POLRMT*) and perilipin 3 (*PLIN3)* in the NEFA group were upregulated (*p* < 0.05). Similarly, compared with the NEFA group, ATP binding cassette subfamily A member 1 (*ABCA1*) in the NEFA + Chol group was upregulated (*p* < 0.05). In addition, compared with the control group, gene expression of peroxisome proliferator-activated receptor α (*PPARα*) in the NEFA group was upregulated (*p* < 0.05). Compared with the control, hepatocytes in the NEFA and NEFA + Chol group had greater protein abundance of NFκB and lesser protein abundance of AKT (Figure 3d–f).

### 3.5. Metabolomics

**Figure 4:**
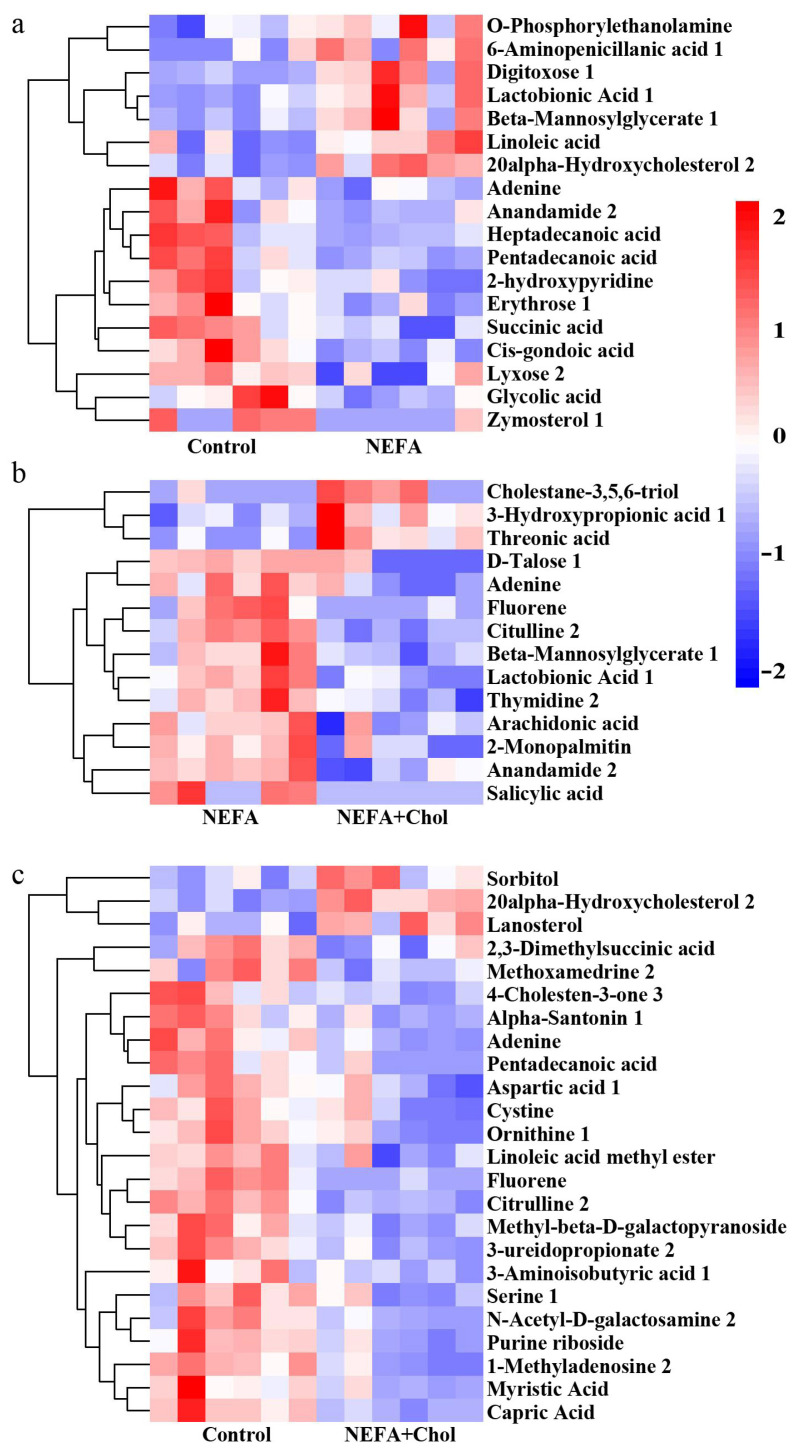
Heatmap of hierarchical clustering analysis of differentially affected metabolites. During a 12 h incubation, isolated hepatocytes were challenged without NEFA (control), 1.2 mM NEFA (c9-18:1, 18:2, 16:0, 18:0, and c9-16:1 at 43.5%, 4.9%, 31.9%, 14.4%, and 5.3% of total NEFA, respectively), or NEFA for 6 h followed by 10 μM choline chloride for another 6 h (NEFA + Chol). Each assay was performed on 6 replicates per treatment group. Differentially affected metabolites in control vs. NEFA (Panel (**a**)), NEFA vs. NEFA + Chol (Panel (**b**)), and control vs. NEFA + Chol (Panel (**c**)) hepatocyte cultures.

**Figure 5:**
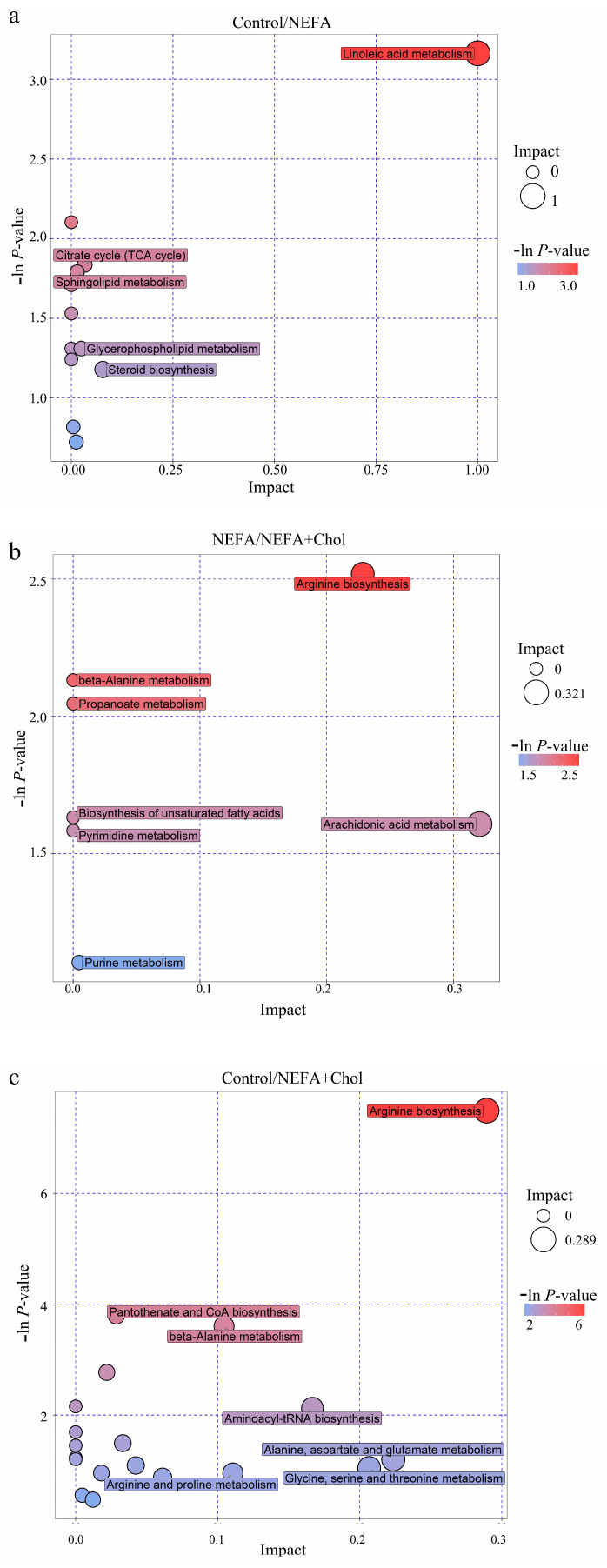
Treemap of pathway analysis using differentially affected metabolites. During a 12 h incubation, isolated hepatocytes were challenged without NEFA (control), 1.2 mM NEFA (c9-18:1, 18:2, 16:0, 18:0, and c9-16:1 at 43.5%, 4.9%, 31.9%, 14.4%, and 5.3% of total NEFA, respectively), or NEFA for 6 h followed by 10 μM choline chloride for another 6 h (NEFA + Chol). Control vs. NEFA (Panel (**a**)), NEFA vs. NEFA + Chol (Panel (**b**)), and control vs. NEFA + Chol (Panel (**c**)). Each assay was performed on 6 replicates per treatment group.

There were 295 metabolites identified in each group in the metabolomic study. A heatmap of hierarchical clustering analysis of differentially regulated metabolites is depicted in Figure 4. Among these, 18 affected metabolites in the comparison control versus NEFA participate in linoleic acid metabolism, glyoxylate and dicarboxylate metabolism, citrate cycle metabolism, sphingolipid metabolism, or steroid hormone biosynthesis (Figure 5a). A total of 14 metabolites in the NEFA versus NEFA + Chol comparison were identified, and participate in the biosynthesis of unsaturated fatty acids, arachidonic acid metabolism, arginine and proline metabolism, or purine metabolism (Figure 5b). A total of 24 metabolites in the control versus NEFA + Chol comparison participate in cysteine and methionine metabolism, aminoacyl-tRNA biosynthesis, glycine, serine, and threonine metabolism, methane metabolism, or steroid biosynthesis (Figure 5c).

### 3.6. Schematic Representation of the Key Regulatory Networks Modulated by Choline Supplementation in NEFA-Challenged Calf Hepatocytes

**Figure 6:**
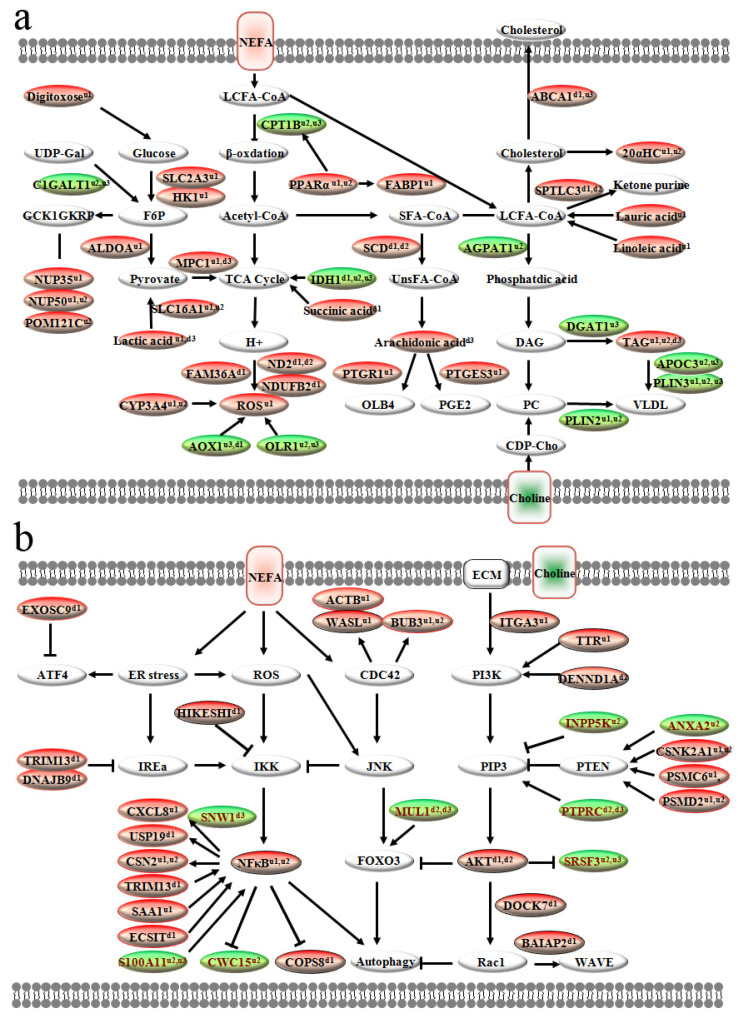
Schematic overview of major differentially abundant proteins and metabolite pathways modulated by choline in high NEFA-challenged calf hepatocytes. (**a**) Lipid metabolism-related signaling pathways regulated by NEFA and choline intervention; (**b**) Endoplasmic reticulum stress and inflammatory signaling pathways affected by NEFA and choline treatment. Hepatocytes were incubated for 12 h with (1) no NEFA (control); (2) 1.2 mM NEFA (composition: c9-18:1 43.5%, 18:2 4.9%, 16:0 31.9%, 18:0 14.4%, c9-16:1 5.3%); or (3) 1.2 mM NEFA for 6 h followed by 10 μM choline chloride for another 6 h (NEFA + Chol). Proteins and metabolites are colored based on their response: red, altered by NEFA vs. control; green, altered by choline vs. NEFA. Arrows indicate positive regulation, T-bars indicate negative regulation, and solid lines indicate direct interactions. Superscripts denote comparisons: u1, higher abundance in NEFA vs. control; u2, higher in NEFA + Chol vs. control; u3, higher in NEFA + Chol vs. NEFA; d1, lower in NEFA vs. control; d2, lower in NEFA + Chol vs. control; d3, lower in NEFA + Chol vs. NEFA.

To integrate the proteomic and metabolomic findings, we constructed a schematic overview of the major regulatory pathways altered by high NEFA treatment and subsequent choline supplementation (Figure 6). Under NEFA challenge (Panel a), key proteins involved in glycolysis (HK1, ALDOA) and lactate production (SLC16A1) were upregulated, while TCA cycle flux was enhanced (IDH1, succinic acid). Concurrently, NEFA promoted β-oxidation via increased CPT1B and PPARα but induced oxidative stress through elevated ROS and dysregulated CYP3A4/AOX1. These metabolic perturbations were partially reversed by choline supplementation, which restored VLDL secretion (DGAT1, APOC3, PLIN3) and promoted fatty acid export.

At the signaling level (Panel b), NEFA exposure activated ER stress (ATF4, IRE1α) and the CDC42/JNK pathway, triggering downstream inflammatory (NF-κB) and autophagic responses. Choline treatment attenuated these stress signals by modulating PI3K/AKT/Rac signaling, reducing autophagy-related proteins (MUL1, COPS8), and suppressing NF-κB-mediated inflammation. Collectively, the integrated network illustrates that choline counteracts NEFA-induced lipotoxicity by restoring lipid export, improving mitochondrial function, and inhibiting stress-related inflammatory and autophagic cascades.

## 4. Discussion

Neonatal calves and periparturient dairy cows differ greatly in hepatic physiological function and metabolic traits, covering lipid metabolism, mitochondrial oxidative activity, and VLDL secretion capacity. Negative energy balance in periparturient cows accelerates adipose lipolysis and massive release of non-esterified fatty acids [14]. Excessive hepatic NEFA accumulation induces mitochondrial dysfunction and oxidative stress [15]. Impaired VLDL assembly and secretion restricts hepatic triglyceride export, leading to lipid deposition and hepatic metabolic disorders [16]. Unlike stress-related metabolic disturbance in periparturient cows, healthy neonatal calves possess an immature but metabolically steady liver. They present faint endogenous lipid mobilization, low basal mitochondrial oxidation, and weak VLDL secretion, without spontaneous lipid overload and oxidative damage [17]. Considering these inherent metabolic discrepancies, exogenous NEFA was used to treat neonatal calf hepatocytes in this study, so as to simulate the hepatocyte model of periparturient dairy cows.

Although several recent studies have enhanced the mechanistic understanding of the role of choline in the context of the physiology of the peripartal cow [18], studying mechanistic aspects of the metabolism of these nutrients in the liver in vivo is very challenging. Although there are drawbacks in using calf hepatocytes to address liver metabolism at the whole-animal level, this model allows for performing a more controlled evaluation of nutrient metabolism beyond the well-studied metabolic pathways [19]. Thus, choline was supplemented after 6 h of NEFA treatment to establish a therapeutic intervention model that mimics clinical scenarios in which fatty liver has already developed. In the current study, high-throughput methods were used to assess the proteome and metabolome spectra in response to NEFA and choline supply.

### 4.1. Responses to a Fatty Acid Challenge

In non-ruminants, under normal conditions, hepatocytes metabolize glucose into pyruvate, which is further metabolized to carbon dioxide in the mitochondria through the TCA cycle and oxidative phosphorylation (OXPHOS) for ATP production [20]. In this research, hepatocytes treated with NEFA showed high glycolysis with high HK1 and ALDOA, which promote glucose to fructose 6-phosphate and finally pyruvate, and high SLC16A1express which regulate transform between pyruvate and lactate. Liver disease, such as non-alcoholic fatty liver disease (NAFLD), is associated with defective mitochondrial OXPHOS and an increase in reactive oxygen species (ROS) production [19]. In addition, progressive hepatocyte damage was characterized by greater abundance of HK1 and ALDOA, and lactate production [21], supporting other data demonstrating that hepatocytes under these conditions increase the rate of glycolysis with consequent lactate production and efflux via the SLC16A1 [22]. Thus, the fact that in the present study hepatocytes treated with NEFA had a greater abundance of SLC16A1 and lactate production also indicated that the rate of glycolysis increased in response to excess NEFA.

Among glycolytic intermediates, fructose 6-phosphate stimulates nuclear pore protein nucleoporin 35 (NUP35), nuclear pore protein nucleoporin 50 (NUP50), and nuclear envelope pore membrane protein (POM121C) to translocate to the nucleus [23] along with the complex of glucokinase (GCK1) and glucokinase regulatory protein (GKRP) [24]. In addition, the greater abundance of MPC1, which cotransports pyruvate from the mitochondrial intermembrane space to the mitochondrial matrix [25], is an example of a low-carbohydrate state in cells treated with NEFA alone. Taken together, the greater abundance in metabolism of carbohydrates (e.g., NUP35) and TCA cycle (e.g., MPC1)-related proteins in hepatocytes challenged with NEFA reinforced the idea of an increase in glycolysis rates and TCA cycle activity due to high levels of fatty acids.

Oxidation of fatty acids and pyruvate in the mitochondrial matrix yields large amounts of NADH. The respiratory electron transport chain couples the NADH to NAD+ to the export of protons from the mitochondrial matrix. In this study, hepatocytes treated with NEFA showed a lesser abundance of respiratory electron transport chain regulators FAM36A, ND2, and NDUFB2. FAM36A is an important factor in the early steps of mitochondrial cytochrome c oxidase assembly that promotes the mitochondrial gene product COX20 and its assembly into the mitochondrial inner membrane CCO complex [26]. ND2 and NDUFB2 participate in mitochondrial Complex I, which utilizes NADH to pump protons out of the mitochondrial matrix. The lesser abundance of FAM36A, ND2, and NDUFB2 in response to NEFA challenge suggested an impairment in the respiratory electron transport chain, e.g., CCO complex and Complex I may have been damaged, likely due to excessive superoxide production and increased oxidative stress [27,28,29]. Under those conditions, liver cells trigger protective programs against ROS-inflicted cell death, rather than mitochondrial metabolism that contributes to the generation of ROS. This biological response corroborates previous work in patients with NAFLD and supports the idea of increased β-oxidation and lactate accumulation in liver cells exposed to high levels of fatty acids [30].

De novo fatty acid synthesis to form TAG and direct re-esterification to form VLDL are the main metabolic pathways of fatty acids in the liver. In this study, hepatocytes treated with NEFA showed significantly higher concentrations of TAG and linoleic acid (LA) and downregulated stearoyl-coenzyme A desaturase 1 (SCD1). SCD1 is the enzyme responsible for catalyzing the endogenous desaturation of saturated fatty acids (SFAs) to the synthesis of monounsaturated fatty acids, i.e., mainly oleate and palmitoleate. These fatty acids can be used as substrates for the synthesis of TAG, cholesterol esters, and phospholipids (PL) [31]. Greater expression of SCD1 in different rodent cell lines supplemented with SFA was associated with SFA incorporation into TAG species. Our recent study showed lower SCD1 mRNA abundance in calf hepatocytes challenged with oleic acid (OA), LA, and palmitoleic acid (POA) compared with palmitic acid (PA) and stearic acid (SA)-treated cells, and this study also showed that SFA causes more severe oxidative and endoplasmic reticulum stress, while unsaturated fatty acids cause marked lipid accumulation [32]. Although SCD1 can prevent liver injury, mostly by partitioning the excess of lipids into monounsaturated fatty acids that can be safely stored in the liver, the lesser abundance of SCD1 in NEFA-challenged hepatocytes suggests reduced cell viability due to induced apoptosis and autophagy as previously reported in non-ruminants [33].

Dysregulation of lipid metabolism, insulin signaling, inflammation, and immune response has an important role in NAFLD pathogenesis. The PI3K/AKT pathway is considered a link between inflammation and NAFLD, and decreased PI3K/AKT-related proteins were observed in NAFLD induced through both high-fat diet and genetic factors [34,35,36]. Within this pathway, the lesser abundance observed in PI3K/AKT regulation factors AKT, DENN domain containing 1A (DENND1A), dedicator of cytokinesis 7 (DOCK7), along with the upregulation in negative regulation factors PTEN, and INPP5K protein (INPP5K), are examples of decreased PI3K/AKT/RAC-related proteins, and indicate increased mitochondrial autophagy during a fatty acid challenge. In humans, DENND1A was reported as an activator of the PI3K/AKT signaling pathway [37]. PI3K plays a key role in glucose and lipid metabolism, and PTEN can dephosphorylate the second messengers generated by the activation of PI3K, thereby downregulating the PI3K downstream signal [38]. In addition, INPP5K has been suggested to negatively regulate PI3K levels. At least in non-ruminants, INPP5K heterozygotes are insulin sensitive with modest resistance to diet-induced obesity, and reinforce a negative role of INPP5K in AKT signaling [39]. A key function of AKT is to activate lipogenesis, in part via the mammalian target of rapamycin complex 1 (mTORC1) signaling complex. The activation of the PI3K/AKT pathway enables RAC family small GTPase 1 (RAC1) to bind to and activate NADPH oxidase, ultimately leading to the production of H2O2, which induces ROS production [40]. To balance the proliferative and survival advantage of moderate ROS levels versus their damaging effect at high concentrations, cells undergo a redox adaptation process through the induction of the antioxidant response [41]. Annexin A2 (ANXA2) plays a key role in protecting cells from ROS-induced death by positively regulating PTEN activity, hence, inhibiting the PI3K/AKT pathway [42]. Furthermore, FOXO3 induces ubiquitylation of AKT through mitochondrial E3 ubiquitin-protein ligase 1 (MUL1) regulation. Thus, the upregulation in PTEN, along with depressed PI3K/AKT/RAC-related proteins in response to NEFA challenge, might be a negative feedback mechanism by NEFA-induced lipotoxicity.

The transcription factor NFκB is activated by different pathogenic stimuli, including growth factors, cytokines, radiation, and oxidative stress. Activation of NFκB controls many genes involved in inflammatory processes, and high levels of NEFA were associated with NFκB activation due to increased oxidative stress [43,44]. AKT positively influences IKK (IκB kinase)-mediated NFκB activation through the downstream activation of mTORC1, while LPS-induced NFκB phosphorylation was unaffected by the inhibitor of PI3K in mouse macrophages [45]. In addition, the activation of the ROS/NFκB axis supported changes in catabolism of eicosanoids and lipid peroxidation status through greater abundance of prostaglandin reductase-1 (PTGR1) in response to NEFA challenge. Thus, we speculate that high levels of NEFA-induced ROS might modulate NFκB activation and its target genes in an attempt to dampen oxidative stress [43,46].

In the present study, NEFA-challenged hepatocytes showed a greater abundance of cell division cycle 42 (CDC42) pathway-related proteins, e.g., actin cytoplasmic (ACTB), neural Wiskott–Aldrich syndrome protein (WASL), and mitotic checkpoint protein BUB3 (BUB3), coupled with lower RAC pathway-related proteins, e.g., DOCK7 and brain-specific angiogenesis inhibitor 1-associated protein 2 (BAIAP2), suggesting an overall increase in cell adhesion damage. CDC42 is known to play important roles in the organization of the cytoskeleton, cell proliferation, cell polarity, cellular transport, and is necessary for the proper regulation of hepatocytes’ lipid metabolism [47]. In rodents, hepatocyte-specific deletion of CDC42 disrupted intracellular localization of ATP-binding cassette subfamily A member 1 (ABCA1) and was associated with delayed liver regeneration after partial hepatectomy [48]. These data, along with the responses in CDC42/JNK pathway-related proteins when hepatocytes were challenged with NEFA, might help explain a cellular overload and increased mitochondrial autophagy.

At least in non-ruminants, CDC42 and RAC1 are major contributors to the SFA-stimulated JNK pathway in hepatocytes [49]. The overexpression of RAC1 and CDC42 in fibroblasts revealed that only a greater abundance of CDC42 induced NFκB. As in our current study, these responses were associated with increased autophagy and glycolytic metabolism, coupled with a marked decrease in mitochondrial activity. Regardless of differences in the cellular type, taken together, these results suggested that CDC42 stimulates JNK more potently. As such, it can be speculated that RAC1 can potently activate both JNK and AKT kinases, and initiate an AKT-dependent (but NFκB-independent) survival mechanism, which is prevalent over the apoptotic signals mediated by JNK [50]. Furthermore, previous studies suggested that NEFA-induced activation of the CDC42/JNK pathway and the ROS/NFκB axis, along with inhibition of PI3K/AKT/RAC1, led to greater mitochondrial autophagy in rodent hepatocytes [51,52,53,54]. Thus, the findings of CDC42 and NFκB-related proteins led us to focus on targets that could play a relevant role in cell autophagy and inflammatory processes.

### 4.2. Responses to Increased Choline Supply

Although the effects of choline supplementation in reducing liver TAG are inconsistent in studies conducted with dairy cattle, those studies that showed beneficial effects associated such responses with an increase in PC synthesis through the CDP–choline pathway [55]. In addition, recent work with dairy cows reported upregulation of apolipoproteins-related genes due to increased post-ruminal choline supply [11,56]. These lipoproteins provide support for enhanced hepatic synthesis of VLDL, which plays a fundamental role in reducing liver TAG accumulation and was underscored as the main explanation for the positive effects of choline supply on liver lipid metabolism [57]. Previous work suggested that synthesis of TAG, rather than being symptomatic of hepatic lipid overload, actually serves a protective role by enhancing lipid droplet formation and alleviating the potential for NEFA to induce a lipotoxic effect [3]. This effect was observed in the present study through greater TAG in hepatocytes incubated with NEFA and NEFA + Chol. However, the lower TAG concentrations in NEFA + Chol compared with NEFA cells can be partly explained by the greater abundance of DGAT1, APOC3, and PLIN3 in choline-supplemented cells.

From a mechanistic standpoint, the fact that DGAT1 exhibits substrate preference for exogenously derived NEFA for TAG synthesis and incorporation into VLDL agrees with the present in vitro results. In the same way, a recent study conducted with murine models associated a greater DGAT1 expression with increased export of liver TAG and underscored the importance of this key metabolic enzyme on lipid metabolism and extra-hepatic fat partitioning [3]. At least in humans, APOC3 is associated with hepatic VLDL assembly/secretion under lipid-rich conditions and is directly linked to a reduction in liver TAG [58]. The greater APOC3 with choline supply suggested an increase in VLDL assembly and corroborates data in peripartal dairy cows receiving rumen-protected choline. Thus, the greater APOC3 in response to increased choline supply was another indication of liver TAG reduction in choline-treated hepatocytes during a NEFA challenge.

Lipid droplets are surrounded by a phospholipid monolayer and coated with unique proteins. Among the perilipin family, perilipin 2 (PLIN2) and PLIN3 are the predominant lipid droplet-associated proteins [19]. PLIN2 has a high affinity for PC, which supports the increase in TAG and VLDL concentrations detected in the NEFA + Chol versus non-challenged cells. Lower levels of PLIN2 result in the formation of smaller lipid droplets [59], which were observed in the control group. Under normal conditions, unlike PLIN2, PLIN3 targets nascent lipid droplets primarily and stimulates lipid storage. However, PLIN3 also participates in the process of lipid droplet dispersion, suggesting that a greater abundance of PLIN3 in the NEFA + Chol cells may have facilitated lipolysis and β-oxidation of fatty acids [60]. Furthermore, a study in knockout mice showed that silencing of MPC1 activity boosted fatty acid oxidation [61], and this behavior seemed to have occurred in choline-treated cells due to the lesser abundance of MPC1in NEFA + Chol versus NEFA cells. Thus, besides reducing lipotoxicity by promoting the elimination of TAG in the form of VLDL, the present data suggested that choline might have enhanced mitochondrial β-oxidation and facilitated fatty acid oxidation as recently reported in vivo.

The enzyme CPT1 catalyzes the conversion of long-chain fatty acyl-CoAs into acylcarnitine for transport into the mitochondrial matrix for β-oxidation to acetyl-CoA, followed by metabolism via the TCA cycle or ketogenesis [62]. Greater CPT1B in the NEFA + Chol compared with the NEFA group suggested an increase in mitochondrial fatty acid degradation, and was consistent with the increased fatty acid mitochondrial oxidation in the liver of cows supplemented with post-ruminal choline [63]. These responses observed due to increased choline supply might be related to an increased availability of methyl donors within hepatocytes as a result of greater choline availability [56]. In rodents, a greater abundance of liver CPT1A gene expression was associated with increased betaine-homocysteine methyltransferase (BHMT) activity and consequent effects on DNA methylation of the PPARα promoter region [64]. As BHMT catalyzes the transfer of a methyl group from betaine to homocysteine to generate Met [65], we speculated that increased availability of Met might have led to changes in DNA methylation and transcriptional regulation. Similar responses with CPT1A upregulation were also observed in human hepatocytes treated with choline [66]. Although BHMT and PPARα effects were not confirmed directly in the present study, modulation of one-carbon metabolism with increased availability of methyl donors (e.g., choline and Met) supports the increase in CPT1A [56].

Integrated analysis of metabolomic and proteomic data revealed coordinated alterations in hepatic metabolic networks. In NEFA-challenged hepatocytes, enhanced glycolysis (elevated HK1, ALDOA, SLC16A1) was accompanied by increased lactate and TCA cycle intermediates, indicating a metabolic shift toward anaerobic metabolism. In addition, previous studies have demonstrated that NEFA exposure enhances mitochondrial autophagy in calf hepatocytes, as evidenced by increased protein abundance of LC3-II and p62 [67]. Meanwhile, NEFA suppresses fatty acid oxidation, promotes excessive ROS production, and elevates mitochondrial membrane potential [68]. Impaired mitochondrial respiratory chain proteins (FAM36A, ND2, NDUFB2) coincided with oxidative stress and activated ROS/NFκB signaling, which further triggered mitochondrial autophagy. Choline supplementation restored lipid homeostasis by enhancing VLDL secretion (DGAT1, APOC3, PLIN3) and mitochondrial β-oxidation (CPT1B), while reducing inflammation and autophagy. These coordinated changes across the proteome and metabolome demonstrate that choline alleviates NEFA-induced lipotoxicity by restoring mitochondrial function, lipid export, and cellular redox balance.

Notably, although calf hepatocytes were widely used to elucidate regulatory mechanisms of hepatic lipid metabolism in adult ruminants [69,70], caution should be exercised when extrapolating findings from 1-day-old calves due to distinct age-related differences in hepatic lipid metabolism. Because there are clear differences in aspects of lipid metabolism as a function of age [17]. Although the current in vitro experimental data indicat abundance e that choline can restore mitochondrial function, lipid output, and cellular redox balance, the actual role of choline in the metabolism of dairy cow liver remains to be determined.

## 5. Conclusions

Data suggested that calf hepatocytes treated with high NEFA concentrations respond by enhancing glycolysis rate and lactate accumulation, along with increased TCA cycle activity. Activation of the CDC42/JNK pathway and ROS/NFκB axis, along with lesser abundance in PI3K/AKT/RAC-related proteins, also indicated increased mitochondrial autophagy and impaired respiratory transport chain in response to NEFA. As observed in some in vivo studies with dairy cows, the increased supply of choline in vitro reduced NEFA-induced hepatocyte TAG accumulation. This response occurred partly through enhanced VLDL secretion, increased mitochondrial β-oxidation, coupled with a lower degree of lactate accumulation, cellular autophagy, and inflammation.

This study improves our understanding of hepatic lipid metabolism disorders in transition dairy cows and offers a theoretical basis for nutritional intervention strategies. The application prospects of these results include optimizing rumen-protected choline supplementation regimens to prevent and alleviate fatty liver and metabolic stress in periparturient dairy cows, thereby improving liver health, metabolic stability, and productive performance in modern dairy farming.

## Figures and Tables

**Table 1:** Subpathway analysis of greater or lesser abundance proteins. During a 12 h incubation, isolated hepatocytes were challenged without NEFA (control), 1.2 mM NEFA (c9-18:1, 18:2, 16:0, 18:0, and c9-16:1 at 43.5%, 4.9%, 31.9%, 14.4%, and 5.3% of total NEFA, respectively), or NEFA for 6 h followed by 10 μM choline chloride for another 6 h (NEFA + Chol). Superscript u1 represents greater abundance in NEFA compared with the control group, u2 represents greater abundance in NEFA + Chol compared with control, u3 represents greater abundance in NEFA + Chol compared with NEFA, d1 represents lesser abundance in NEFA compared with control, d2 represents lesser abundance in NEFA + Chol compared with control, and d3 represents lesser abundance in NEFA + Chol compared with NEFA. Bold indicates that gene expression of the protein was verified by qRT-PCR.

Pathway	Subpathway	Up-Regulated and Down-Regulated Proteins
Metabolism (*p* = 1.38 × 10^−9^)	Metabolism of lipids	PTGR1^u1^, FABP1^u1^, **PPT1**^u2^, **CSNK2A1**^u1,u2^, PLIN2^u1,u2^, **AGPAT1**^u2^, **INPP5K**^u2^, **CPT1B**^u2,u3^, **PLIN3**^u2,u3^, DGAT1^u3^, ABCA1^d1^, **SCD1**^d1,d2^, **SPTLC3**^d1,d2^
	Metabolism of carbohydrates	ALDOA^u1^, **NUP35**^u1^, HK1^u1^, NUP50^u1,u2^, POM121C^u2^
	The citric acid cycle and respiratory electron transport	**SLC16A1**^u1,u2^, **MPC1**^u1,d3^, **FAM36A**^d1^, NDUFB2^d1^, ND2^d1,d2^
	Metabolism of amino acids and derivatives	**PSMC6**^u1^, **PSMD2**^u1,u2^, LARS^u2,u3^, **RPS10**^d1^, PRODH2^d2^
	Metabolism of vitamins and cofactors	SLC2A3^u1^, APOC3^u2,u3^, AOX1^u3,d1^, **ABCD4**^d2^
	Biological oxidations	**PTGES3**^u1^, CYP3A4^u1,u2^, ABHD14B^u2^, **PODXL**^d1^
	Metabolism of nitric oxide	WASL^u1^
	Regulation of insulin secretion	SLC2A3^u1^
Metabolism of proteins (*p* = 1.97 × 10^−5^)	Regulation of IGF transport and uptake by IGFPBs	TGOLN2^u1,u2^, KNG1^d1^
	tRNA Aminoacylation	LARS^u2,u3^, **WARS**^d1,d2^
	Amyloid fiber formation	SAA1^u1^
	Unfolded protein response (UPR)	**CXCL8**^u1^, DNAJB9^d1^, EXOSC9^d1^
	Chaperonin-mediated protein folding	ACTB^u1^, **CSNK2A1**^u1,u2^
	Deubiquitination	**PSMC6**^u1^, **PSMD2**^u1,u2^, USP19^d1^, MUL1^d2,d3^
	Mitochondrial translation elongation	MRPL2^u1,u2^, MRPS30^d1^, MRPL49^u3^
	RAB geranylgeranylation	RAB8B^u2,u3^, RAB22A^d1^
	SRP-dependent cotranslational protein targeting to the membrane	SRP54^u2,u3^
	Asparagine N-linked glycosylation	TRIM13^d1^, **ANK3**^d3^
	Neddylation	COPS8^d1^
	SUMO E3 ligases SUMOylate target proteins	**NUP35**^u1^, NUP50^u1,u2^, POM121C^u2^
	O-linked glycosylation (Homo sapiens)	C1GALT1^u2,u3^
	Eukaryotic translation initiation	EIF2S2^u3^, **RPS10**^d1^, EIF3I^d2^
Metabolism of RNA (*p* = 8.25 × 10^−5^)	Deadenylation-dependent mRNA decay	DDX6^d1^, EXOSC9^d1^
	Metabolism of non-coding RNA	**NUP35**^u1^, NUP50^u1,u2^, POM121C^u1,u2^
	Regulation of mRNA stability by proteins that bind AU-rich elements	**PSMC6**^u1^, **PSMD2**^u1,u2^, TNPO1^u1,u3^
	Transport of mature transcript to the cytoplasm	SRSF3^u2,u3^
	Nonsense-mediated decay	***RPS10*** ^d1^
	mRNA Splicing	CWC15^u2^, PUF60^d1^, SNW1^d3^
	rRNA processing in the nucleus and cytosol	NOL11^u3^
Signal transduction (*p* = 0.412)	Signaling by receptor tyrosine kinases	ITGA3^u1^, ACTB^u1^, CSN2^u1,u2^, **ATP6V1H^u^^3^**, **DOCK7**^d1^, BAIAP2^d1^
	PIP3 activates AKT signaling	**PSMC6**^u1^, **CSNK2A1**^u1,u2^, **PSMD2**^u1,u2^
	Signaling by TGF-beta family members	FKBP1A^u1^
	Signaling by nuclear receptors	**PTGES3**^u1^, **CPT1B**^u2,u3^
	GPCR downstream signaling	**CXCL8**^u1^, APOC3^u2,u3^, HCRTR2^d3^
	RHO GTPase Effectors	BUB3^u1,u2^, MYH11^u2^
	TNF signaling	SPPL2A^u2,u3^, ADAM17^d3^
	p75 NTR receptor-mediated signaling	ARHGEF2^u1^
	Pre-NOTCH expression and processing	SNW1^d3^
Immune system (*p* = 0.025)	Signaling by Interleukins	**PSMC6**^u1^, **CXCL8**^u1^, **NUP35**^u1^, **PSMD2**^u1,u2^, NUP50^u1,u2^, POM121C^u2^
	Neutrophil degranulation	SLC2A3^u1^, TTR^u1^, **METTL7A**^u2^, ANXA2^u2^, **OLR1**^u2,u3^, S100A11^u2,u3^, ORM1^d1,d2^, IDH1^d1,u2,u3^, **PTPRC**^d2,d3^
	Toll-like receptor 10 (TLR10) cascade	ECSIT^d1^
	Interferon signaling	ISG20^u2^, ISG15^d3^
	Fc gamma receptor (FCGR) dependent phagocytosis	ACTB^u1^, BAIAP2^d1^
	Cytokine signaling in the immune system	ADAM17^d3^
	Complement cascade	C8A^u3^
	ROS, RNS production in phagocytes	**ATP6V1H** ^u3^
	Signaling by the B cell receptor (BCR)	FKBP1A^u1^
	Class I MHC-mediated antigen processing and presentation	B2M^d3^
Gene expression (*p* = 0.232)	RNA polymerase II transcription	INTS1^u1^, TAF9B^u1^, **PSMC6**^u1^, **PSMD2**^u1,u2^, **FAM36A**^d1^, SNW1^d3^, **COBRA1**^d3^
	RNA polymerase III transcription	SSB^d1,d2,d3^
	Transcriptional activation of mitochondrial biogenesis	**POLRMT** ^d2,d3^
	Transcriptional regulation by small RNAs	**NUP35**^u1^, NUP50^u1,u2^, POM121C^u2^
	Negative epigenetic regulation of rRNA expression	POLR1A^d1,d2^
Disease (*p* = 0.871)	HIV infection	**NUP35**^u1^, **PSMC6**^u1^, TAF9B^u1^, **PSMD2**^u1,u2^, NUP50^u1,u2^, POM121C^u2^, PDCD6IP^u2^, **COBRA1**^d3^, B2M^d3^
	Signaling by NOTCH1 in cancer	ADAM17^d3^, SNW1^d3^
	Signaling by the TGF-beta receptor complex in cancer	FKBP1A^u1^
	Influenza infection	**RPS10**^d1^, ISG15^d3^
Developmental biology (*p* = 0.178)	L1CAM interactions	ACTB^u1^, **CSNK2A1**^u1,u2^, **ANK3**^d3^
	Signaling by ROBO receptors	**PSMC6**^u1^, **PSMD2**^u1,u2^, **RPS10**^d1^
	Semaphorin interactions	*MYH11*^u2^, **PTPRC**^u2,u3^
	Regulation of beta-cell development	SLC2A3^u1^, SNW1^d3^
	Keratin filaments bind cell–cell adhesion complexes	KRT80^d1^
	Netrin-1 signaling	WASL^u1^
Transport of small molecules (*p* = 0.228)	SLC-mediated transmembrane transport	SLC2A3^u1^, **SLC16A1**^u1,u2^
	ABC-family proteins mediated transport	**PSMC6**^u1^, **PSMD2**^u1,u2^, EIF2S2^u3^
	HDL assembly	ABCA1^d1^
	Ion channel transport	**ATP6V1H**^u3^, CLCN7^d2^
Cell cycle (*p* = 0.089)	M Phase	**PSMC6**^u1^, **NUP35**^u1^, **PSMD2**^u1,u2^, BUB3^u1,u2^, **CSNK2A1**^u1,u2^, NUP50^u1,u2^, POM121C^u2^
Cellular responses to external stimuli (*p* = 0.159)	Oxidative stress-induced senescence	**CXCL8**^u1^, **NUP35**^u1^, NUP50^u1,u2^, POM121C^u2^
	Cellular response to heat stress	**PTGES3**^u1^, **HIKESHI**^d1^
	Cellular response to hypoxia	**PSMC6**^u1^, **PSMD2**^u1,u2^
Hemostasis (*p* = 0.529)	Cell surface interactions at the vascular wall	ITGA3^u1^, **SLC16A1**^u1,u2^
	Factors involved in megakaryocyte development and platelet production	ACTB^u1^, AKAP2^u2^, **DOCK7**^d1^
	Response to elevated platelet cytosolic Ca2+	TMX3^d2^
Cell–cell communication (*p* = 0.046)	Adherens junctions interactions	ACTB^u1^, **ACTN2**^u1^
	Nephrin binds CASK	CASK^d1,d2^
Vesicle-mediated transport (*p* = 0.946)	Membrane trafficking	ACTB^u1^, CYTH1^d1,d2,d3^, **DENND1A**^d2^
Muscle contraction (*p* = 0.146)	Calcium binds to troponin-C	ACTA1^u1^, MYH11^u2^
	Smooth muscle contraction	ANXA6^u2,u3^

## Data Availability

The original contributions presented in this study are included in the article. Further inquiries can be directed to the corresponding author(s).

## Appendix A

**Table A1:** Sequences of primers used for real-time PCR amplification.

Gene	5′-3′	Primer Sequences	Length (bp)
*ABCA1*	Forward	GGACATGTGCAACTACGTGG	157
	Reverse	TGATGGACCACCCATACAGC	
			
*ABCD4*	Forward	CTCAGCAGCCTAGTCAGCAAGAAC	92
	Reverse	TCGGAGAGCGTGGTGCAGAG	
			
*ACTB*	Forward	CTGGCACCACACCTTCTACAACG	103
	Reverse	CATCTTCTCACGGTTGGCCTTGG	
			
*ACTN2*	Forward	GACGCTGTGAATGTTAATGATC	137
	Reverse	TCCAGGTGAAGTTGATCGATG	
			
*AGPAT1*	Forward	CTGCTGCTGCTGCTCTTCCTG	200
	Reverse	ATGTGGAGTAGCATCAGACGCAAG	
			
*ANK3*	Forward	GACAGGCGACACGGACAAGTATC	166
	Reverse	GGCGTAGGAGCTTCGGTTCAAG	
			
*APOC3*	Forward	CTGCTCCTTCTTGCTGCCTTCC	125
	Reverse	GCATCCTTGGCGGTCTTGGTG	
			
*ATP6V1H*	Forward	GAACCACATGCACCACGAGGAC	116
	Reverse	GCGGCTGTTCAGACTGGAGTTG	
			
*AXO1*	Forward	TGCCACTGTCCAGTATCCACCTC	92
	Reverse	GTTGAGATCAGCCACTACGGAACC	
			
*COBRA1*	Forward	TCTTCGACGGCTTCCTCCTCAC	160
	Reverse	TTCACCGCCTCTCCGCTCTG	
			
*CPT1B*	Forward	ACACATCTACCTGTCCGTGATCA	72
	Reverse	CCCCTGAGGATGCCATTCT	
			
*CSNK2A1*	Forward	TCTCCTGATGCCTGAGCAGAGG	116
	Reverse	TCCACAAGCAACGGTTCAGACAC	
			
*CXCL8*	Forward	TGCCTGTTGAACTGCGCCTTG	102
	Reverse	AGTGCTTCCACATGTCCTCACATC	
			
*DENND1A*	Forward	ACAGCCTCACAGTTAGCCAAGTTG	99
	Reverse	CTCCTGAAGATAAGCGGCAGAACC	
			
*DOCK7*	Forward	TTCCGTACTGTCTCCTGAGGTTCC	188
	Reverse	TTCGCATGTCTCCAATTCGGACTG	
			
*FAM36A*	Forward	ATCTGTTGTGGCTGGCCTTG	115
	Reverse	CCTACAGTGGAACCAGCATCC	
			
*HIKESHI*	Forward	AATCTGGAGAAGGAAGCCAACACC	143
	Reverse	AACTGAGGACACAGCAGCATTACC	
			
*INPP5K*	Forward	TGTAGTTGTTGCGGACAGGTTGAG	151
	Reverse	AAGCCTGACTTATGAGCAGAAGCC	
			
*METTL7A*	Forward	GGTGCTCTGCTCGGTGAAGAAC	115
	Reverse	CCAGGTGGAAGGCTTATCTGCTAC	
			
*MPC1*	Forward	ACGCCACCAATGAAGTAGCACAG	150
	Reverse	GGAATCCGTGACTCGGCAACAG	
			
*NUP35*	Forward	CTCTCGCCTTCACACCACCAATC	99
	Reverse	TGTACGCTGTAGCCAGAGGTCTC	
			
*NUP50*	Forward	GGCAGCAACTGAGACCAAGGC	131
	Reverse	AAGCAGCACCAGAGGAGGAAGG	
			
*OLR1*	Forward	CCCAGGGAAATGCCTGCTAC	207
	Reverse	GCTGTGACCTTGAGTTAGGCA	
			
*PLIN3*	Forward	GAGCGGGGTGGACACAGTGC	123
	Reverse	CAAGGGATGTGGCGAGGCGG	
			
*PODXL*	Forward	CACCACGCTGCTGGCATCAC	188
	Reverse	GGAACACAGGCAGGCAGACAAG	
			
*POLRMT*	Forward	CCGTGGAGTCCGTGGAGAAGG	190
	Reverse	TCACTGAGCAGGCACAGGTAGG	
			
*PPARα*	Forward	AATGCAGGAGGGTATTGTGC	162
	Reverse	TCCGACTCCGTCTTCTTGAT	
			
*PPT1*	Forward	ATACCGCAACCACAGCATCTTCC	189
	Reverse	GGAATGGTCTCCTTGGCTTGGC	
			
*PSMC6*	Forward	AACACTCTTGGCACGAGCTGTG	187
	Reverse	CCGGCGACCACCAATAGCATC	
			
*PSMD2*	Forward	TCCACAACAGCAACACCATGAGAC	141
	Reverse	TCACACCTGCCACCTCCATACTG	
			
*PTEN*	Forward	GGCAGCCGTTCCGAGGATTATTC	182
	Reverse	GACTTGGCGGTGGCTGATGC	
			
*PTGES3*	Forward	TCCATGATGCTGTTGAGGTCC	123
	Reverse	TCCCTAAGCCAGAGAGCTTCC	
			
*PTPRC*	Forward	CGAGCAAGGAGAGTGAGCAAGAC	172
	Forward	ACCATCTGCCAGAAGTCACCAATG	
			
*RPS10*	Reverse	AGCCGACAGAGACACCTACAGAC	141
	Forward	CAACTTCACTGAGGTGGCTGACC	
			
*SCD*	Reverse	TGCCCACCACAAGTTTTCAG	80
	Forward	GCCAACCCACGTGAGAGAAG	
			
*SLC16A1*	Reverse	ATGGTGGAGGTCCTATCAGCAGTG	110
	Forward	ACAGAAGGAAGCAGCAATCAAGCC	
			
*SPTLCS*	Reverse	CCTGAACGTGGAAGCTGCTATGG	158
	Forward	TCCTTATGGTCGCGCCTGAGAG	
			
*WARS*	Reverse	CAGACCAAGATGAGTGCCAGTGAC	96
	Forward	GGAGAAGGCGTGCTTGTTGACC	
			
*β-actin*	Reverse	GCCCTGAGGCTCTCTTCCA	101
	Forward	GCGGATGTCGACGTCACA	
